# Kearns–Sayre syndrome with a novel large-scale deletion: a case report

**DOI:** 10.1186/s12886-021-02224-7

**Published:** 2022-01-24

**Authors:** Qin Zhu, Chunwen Chen, Jingyan Yao

**Affiliations:** Department of Ophthalmology, the First Affiliated Hospital of Soochow University, 188 Shizi Sreet, Suzhou, 215006 PR China

**Keywords:** Kearns–Sayre syndrome, Large-scale deletion, OCTA

## Abstract

**Background:**

Kearns–Sayre syndrome (KSS) is a rare, multisystem mitochondrial encephalomyopathy. We report a case of KSS with a novel 7.6-kb deletion as assessed through a long-range polymerase chain reaction (PCR) study in the blood. In addition, optical coherence tomography angiography (OCTA) confirmed deep retinal capillary atrophy for the first time.

**Case presentation:**

A 13-year-old patient presented with progressive vision loss and difficulty with eye opening and was diagnosed with progressive external ophthalmoplegia and retinitis pigmentosa (RP). The patient also experienced heart block, vestibular dysfunction, growth retardation and multiple demyelinating lesions. A long-range PCR study in the blood revealed a large-scale Chrm: 6341–13,993 deletion, which was first reported and broadened the genetic spectrum of this disease. The patient underwent complete ophthalmic examination, medical history review and gene detection, resulting in a confirmation of the diagnosis of KSS. The patient was given a pair of applicable glasses to wear and was followed up every 3 months. An implantable pacemaker was also installed based on the advice of the physician.

**Conclusions:**

We reported a novel large-scale deletion in the mitochondrial DNA of KSS, and OCTA was used for the first time to confirm deep retinal capillary atrophy. Furthermore, because ophthalmic symptoms are often the primary manifestation of KSS, the relationship between ophthalmology and mitochondrial diseases should be emphasised.

## Background

Mitochondrial DNA (mtDNA) deficiency is a neuromuscular disorder caused by a genetic defect in mtDNA. Mitochondrial encephalomyopathy is a group of diseases that result from mitochondrial dysfunction involving the central nervous system, endocrine system, extraocular muscle, myocardium, skeletal muscle and other multiple systems. Kearns–Sayre syndrome (KSS) is a rare type of mitochondrial encephalomyopathy that was first described in 1958 and characterised by progressive external ophthalmoplegia and retinitis pigmentosa (RP). Patients usually present before 20 years of age and exhibit additional signs, including heart block, cerebellar ataxia and protein concentration of cerebrospinal fluid > 100 mg/dL [1].

Based on a demographic study of large-scale mtDNA deletions, the prevalence of KSS is estimated to be 1 to 3/100,000 [2]. It was reported that 89% of patients with KSS have external ophthalmoplegia and 86% have ptosis [3]; thus, patients who have one of above-mentioned symptoms are easily misdiagnosed as having a simple ocular disease. Spontaneous mtDNA rearrangement, including deletion, mutation and duplication has been identified as the major cause of KSS [4]. Over the past few decades, a variety of mtDNA rearrangements have been found in different cases of KSS [5]. However, further exploration of the mtDNA mutation or deletion in KSS might be beneficial for understanding the mechanism and developing a treatment for KSS as well as other mitochondrial disorders.

We report a case of KSS with a 7652-bp deletion from nucleotides 6341 to 13,993 of the mtDNA. This novel deletion has not been previously described. Optical coherence tomography angiography (OCTA) first confirmed and quantified the great atrophy of the deep retinal capillary. In this report, we describe our clinical observation and review the literature to improve our understanding of the disease and avoid misdiagnosis. Our study enriched the deletion spectrum of KSS and increased the understanding of KSS.

## Case presentation

**Fig. 1:**
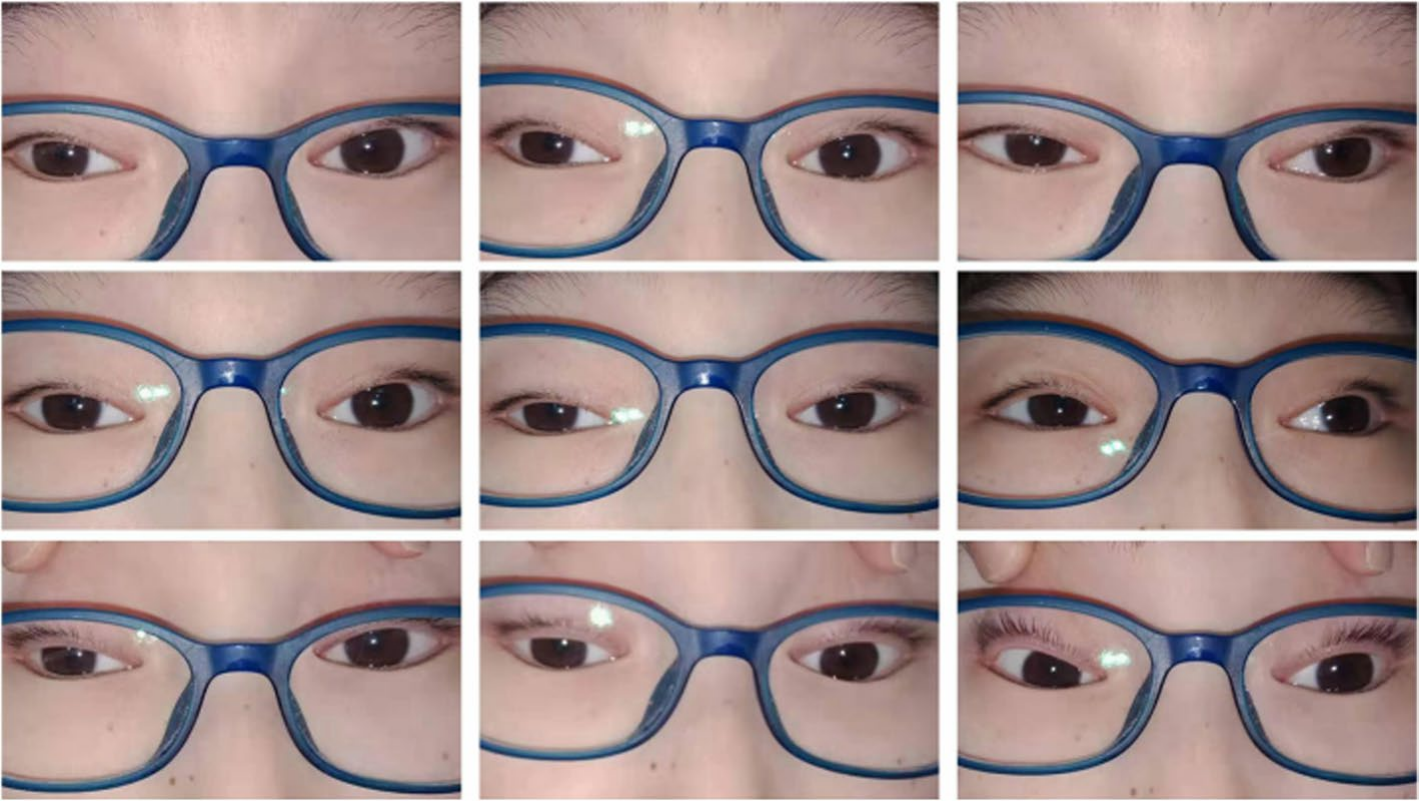
Approximately 5° exotropia was found based on the Hirschberg test, and movements of both eyes were restricted in all gaze directions. Because of weakness in the levator palpebrae muscle, bilateral ptosis was slight and the upper eyelid always covered the upper third of the pupil when looking ahead

**Fig. 2:**
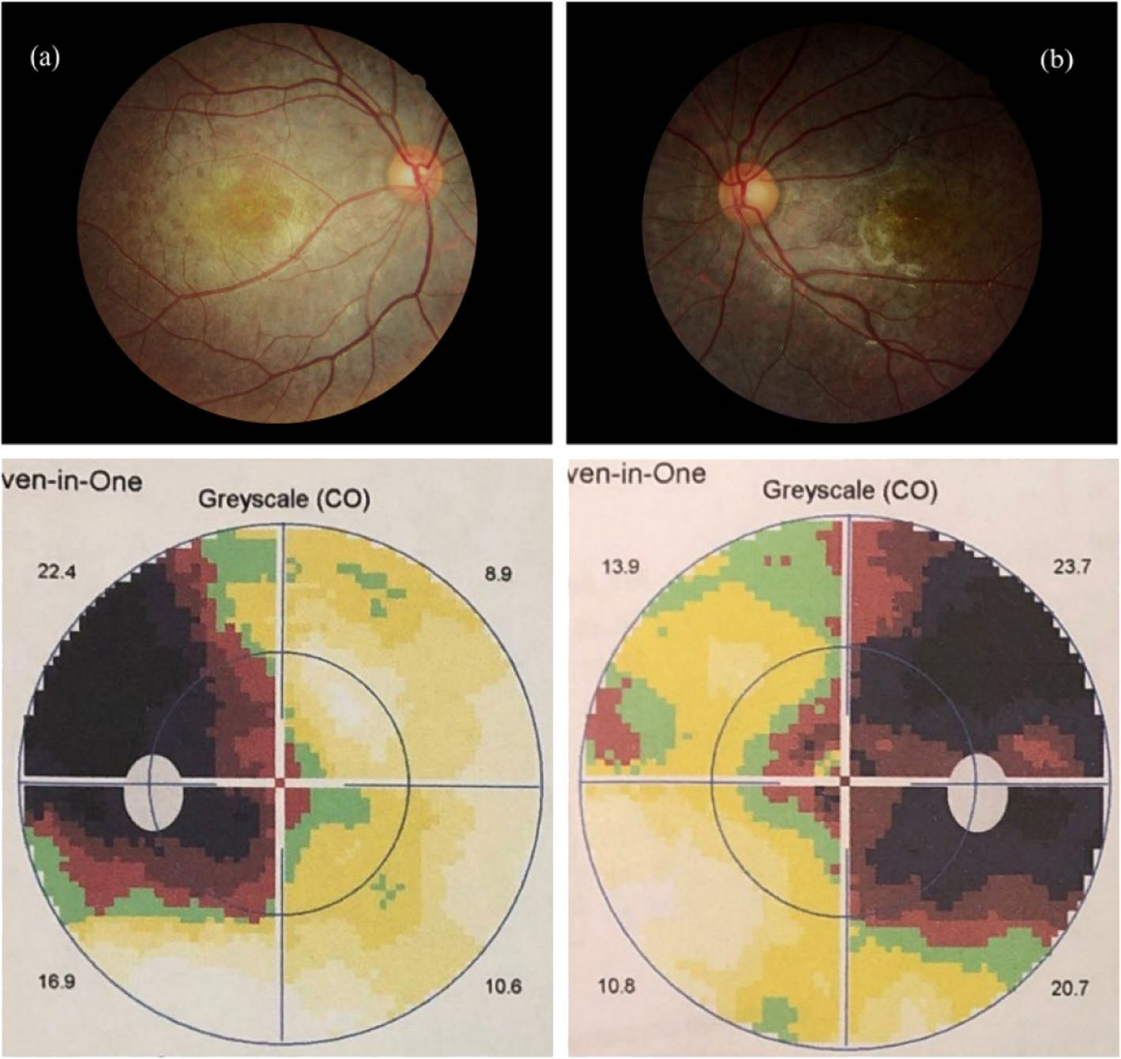
Color fundus photography (**a**, **b**) showed hyperpigmentation of both eyes, and binocular perimetric examination (**c**, **d**) revealed a superior temporal visual field defect and centripetal reduction in both eyes

**Fig. 3:**
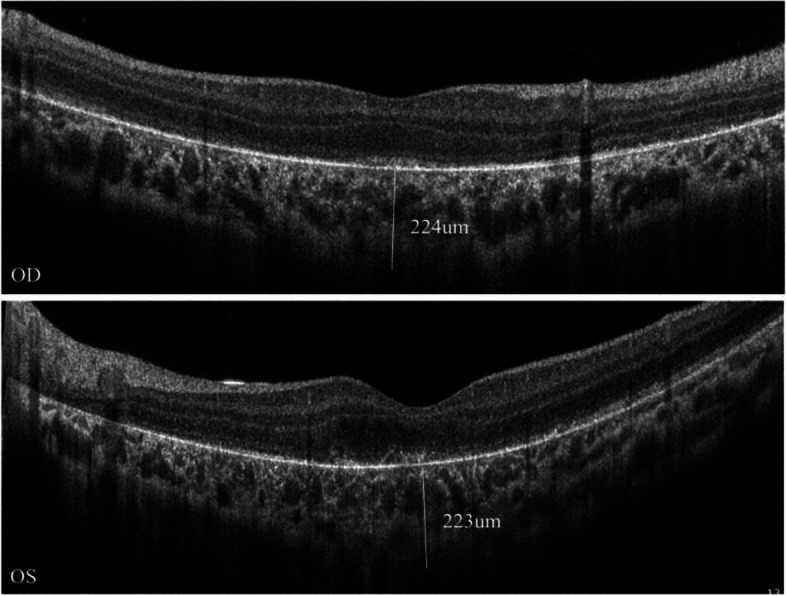
Macular spectral-domain optical coherence tomography revealed an interruption in the ellipsoid band, and several hyperreflective deposits above the retinal pigment epithelium were remarkable. The average subfoveal choroidal thickness (Cht) in both eyes was 223 μm (LE) and 224 μm (RE), respectively

**Fig. 4:**
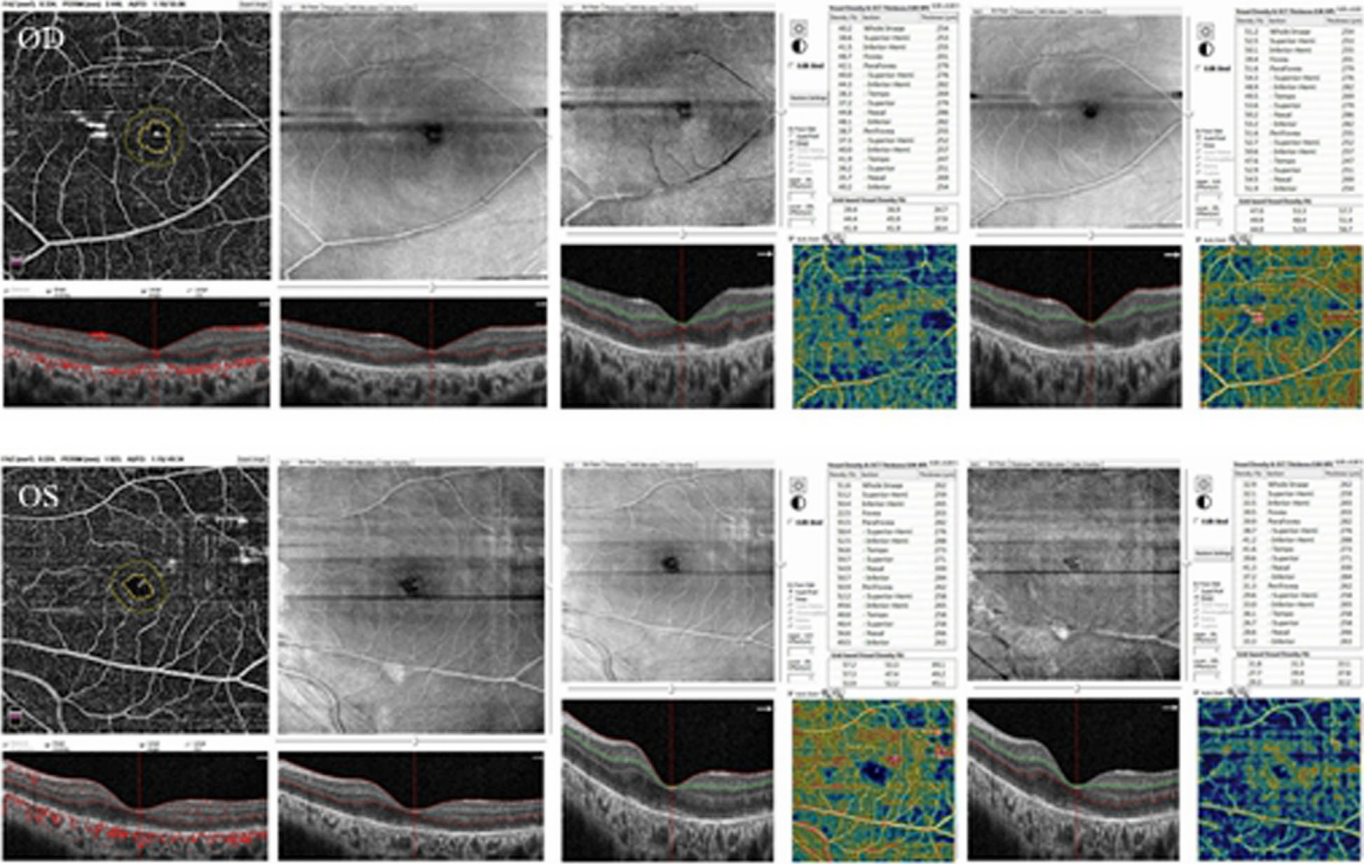
OCTA showed that the deep retinal capillary density decreased and the choroid microvessels atrophied greatly, but foveal area zone and superficial retinal capillary changed insignificantly

A 13-year-old boy presented to our hospital with progressive vision loss and difficulty with opening his eyes. Best-corrected visual acuity was LogMAR 0.3 in the right eye (RE) and LogMAR 0.3 in the left eye (LE), cycloplegic refraction revealed + 0.50 + − 1.50 × 180 (RE) and + 1.00 × 95 (LE). Intraocular pressure was approximately 18 mmHg (RE) and 17 mmHg (LE). Approximately 5° exotropia was found based on the Hirschberg test, and movements of both eyes were restricted in all gaze directions. Because of weakness in the levator palpebrae muscle, bilateral ptosis was slight and the upper eyelid always covered the upper third of the pupil when looking ahead (Fig. 1). Color fundus photography showed hyperpigmentation of both eyes, and binocular perimetric examination (Octopus900, Switzerland) revealed a superior temporal visual field defect and centripetal reduction in both eyes (Fig. 2). Macular spectral-domain optical coherence tomography (Optovue, Fremont, CA, USA) revealed an interruption in the ellipsoid band, and several hyperreflective deposits above the retinal pigment epithelium were remarkable. The average subfoveal choroidal thickness (Cht) in both eyes was 223 μm (LE) and 224 μm (RE), respectively, as measured with built-in software (Fig. 3). OCTA showed that the deep retinal capillary density decreased and the choroid microvessels atrophied greatly, but foveal area zone and superficial retinal capillary changed insignificantly (Fig. 4). The parents denied a history of preterm birth. Family history was negative for individuals with similar symptoms or early-onset vision loss. Genetic testing of the boy and his mother were recommended.

**Fig. 5:**
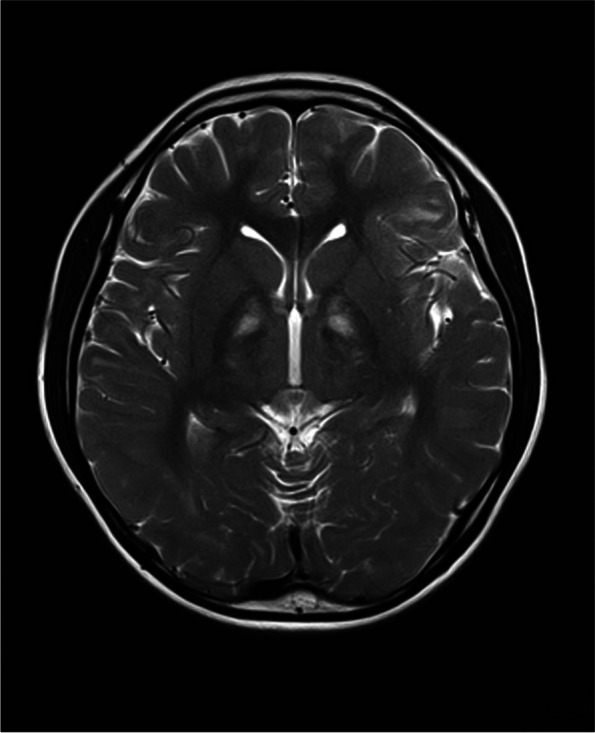
Brain MRI indicated diffuse demyelinating lesions in the white matter of both cerebral hemispheres and the inner capsule, middle cerebral crura, pons and medulla oblongata

The child also complained of dizziness and fatigue, and he had missed school for a long time. From the time of birth, his height and weight had been lower than that of his peers. Currently, his body max index is 14.17 (138 cm tall and 27 kg weight), which is significantly inferior to that of healthy children. He had been diagnosed with growth retardation, and growth hormone therapy was administered. Findings from echocardiography were unremarkable, however, 24-h ambulatory electrocardiogram revealed intermittent first-degree atrioventricular block and partial second-degree type I atrioventricular block. Brain magnetic resonance imaging (MRI) indicated diffuse demyelinating lesions in the white matter of both cerebral hemispheres and the inner capsule, middle cerebral crura, pons and medulla oblongata (Fig. 5).

**Fig. 6:**
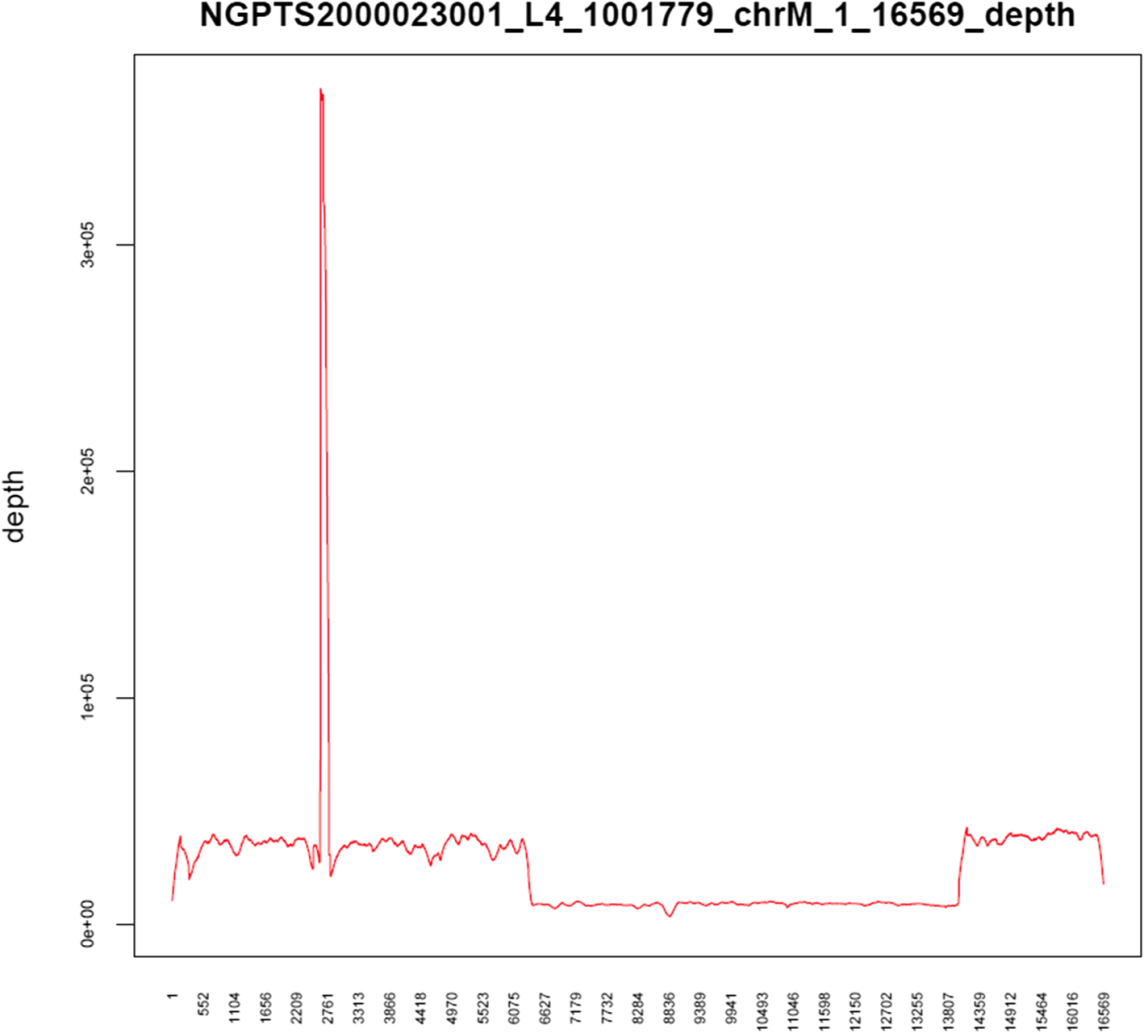
The long-range PCR of the blood samples revealed that the deletion started at nucleotide position 6341 and ended at 13993, indicating a deletion of 7652 bp

Long-range polymerase chain reaction (PCR) was performed on blood samples obtained from both the child and his mother. Specific PCR primers, mtDNA-F: CCGCACAAGAGTGCTACTCTCCTC and mtDNA-R: GATATTGATTTCACGGAGGATGGTG were used to amplify mitochondria in the blood, and a DNA library was constructed using full-length PCR products as a template. Illumina NovaSeq high-throughput sequencing was then performed on the obtained DNA library, and data reading and bioinformatics analysis were performed after evaluating using Sequence Control Software [6]. The long-range PCR of the blood samples revealed that the deletion started at nucleotide position 6341 and ended at 13993, indicating a deletion of 7652 bp (Fig. 6); no abnormality was noted in the mother’s genes.

Complete evidence obtained from ocular examination and evaluation of other organs as well as from gene detection supported the final diagnosis of KSS. After we confirmed the patient’s diagnosis, we provided a pair of applicable glasses after optometry and recommended a follow-up every 3 months. To the best of our knowledge, the physicians provided the appropriate medication, and the patient had an implantable pacemaker installed.

## Discussion and conclusions

A 13-year-old boy complained of blurred vision, eye-opening difficulty and restriction of eye movement. Complete ophthalmological examination revealed remarkable RP, progressive external ophthalmoplegia and ptosis, as well as other systematic symptoms, such as heart block, growth retardation and diffuse demyelinating lesions. The most important findings was obtained from long-range PCR in blood, which demonstrated a 7652-bp deletion from nucleotides 6341–13,993 and enabled the definite diagnosis of KSS.

Mitochondria have an independent DNA and protein synthesis system, and they perform many crucial tasks, but the most significant is the encoding of essential components of the mitochondrial respiratory chain, which drives the synthesis of adenosine triphosphate. An animal study revealed that mitochondria play an important role on the physiology, phenotype and longevity of organisms [7], suggesting that the equal effect occurs in humans. It is estimated that there are thousands of mitochondria per cell in people [8]. To be specific, mitochondria account for about 80% of the volume of extraocular muscles cells [9]. Thus, mitochondrial diseases often invade the eyes, and KSS is accompanied by prominent ocular manifestations.

Because ophthalmoplegia is often symmetrical, patients are not always aware of eye-movement restriction and diplopia, although the earliest complaint is typically difficulty reading because the impaired convergence of bilateral medial rectus weakness. Although our patient presented with symmetrical alteration of ocular motility, RP and palpebral ptosis, this is not always absolute in others. Ahmad et al. reported atypical KSS with a hypopigmented patch in the RE but normal in the LE [10]. The irregular distribution of deleted mtDNA in daughter cells accounts for the tissue variability of clinical pictures presented in mitochondrial disorders [8], so the clinical manifestations of a mitochondrial disease tend to be inscrutable. Interestingly, the pigmentation of KSS is characterised by a diffuse depigmentation of the retinal pigment epithelium (RPE) and described as unique with salt-pepper or moth-eaten appearance [10], which was remarkable in our patient. We measured the average subfoveal Cht of both eyes using built-in software, with the LE and the RE being 223 μm and 224 μm, respectively. However, in an OCT-based study of myopia in Chinese youth, the mean subfoveal Cht was 303.08 ± 76.87 μm [11], and it could be distinguished from the OCT image that it resulted from atrophy of the choriocapillaris layer. Prior research also demonstrated that the choroidal vascularity index, which reflects middle and large choroidal vascularity, has recently shown a downward trend in PR eyes [12]. In OCTA, the foveal avascular zone of the patient’s eyes were 0.334 and 0.224, respectively, and the superior vessel density was 51.2 and 51.6%, respectively, which were similar to the results reported in previous studies [13]. However, there was a marked reduction in deep retinal capillary density as compared with the normal population (62.9% ± 3.5%) in the parafovea [14]. Alvaro Ortiz et al. also discovered a disorder of the deep capillary system in a patient with KSS, which was consistent with our findings; however, the patient in the previous study maintained a normal vascular pattern of choriocapillaries [15]. Thus, this is the first study of the discovery of choriocapillaris layer atrophy through OCTA. At the same time, OCTA has been widely used as an noninvasive technique, and it may gradually be used as an auxiliary method for fluorescein fundus angiography (FFA) prior to diagnosis, particularly in certain situations [16]. We speculate that OCTA could 1 day be used as an option to assess the severity and prognosis of KSS, b ut this still requires confirmation through additional studies. Reynold Spector et al. reported that KSS interfered with the functions of the critical choroid plexus and is one of the causes of generalised choroid plexus failure [17]. Nevertheless, the results of the eye examinations might provide guidance for the diagnosis and treatment of KSS.

Since KSS was first reported, a growing number of deletions in mtDNA at different positions and sizes responsible for this disease have been recognised. It has been reported that more than 90% of KSS tissues have a large deletion of mtDNA fragment [18]. Th e molecular pathological era of mitochondrial disease research has opened since Holt et al. detected the deletion of pathogenic large fragments of mtDNA in KSS patients in 1988 [19], and genetic testing has served as the gold standard for diagnosis [20]. The size of the long-range deletions described in the literature fluctuate from 2 to 10 kb, and the size and location of the mitochondrial deletions vary from individual to individual. However, studies have reported a ‘common deletion’ of 4.9 kb (4977 bp; m.8470–13446del) between ATPase 8 and ND5 genes in one-third of KSS patients [21, 22]. In our study, the long-range PCR study identified a 7652-bp deletion from nucleotides 6341 to 13,993 of the mtDNA, which is accepted as the immediate cause of our patient’s multisystem mitochondrial disorder. Several 7.6-kp deletions have been reported in MITOMAP, including 7647 bp (m.6341–13989del), 7663 bp (m. 6330–13994del) and 7664 bp (m.6329–13994del), which have the same starting point or a similar size of deletion as reported in our current case (http://mitomap.org/MITOMAP). The novel large-scale deletion supports the definite diagnosis of KSS, and to our knowledge, this is the first case to report it. At the same time, because of the disparity of mtDNA rearrangement in additional populations, the constant advances of biochemical markers and expansion of genotypes of mitochondrial disorders pose a challenge. In other words, the analysis mtDNA rearrangement not only is crucial for broadening the alterant spectrum of disease but also vital to acknowledge the connection between mtDNA abnormality and clinical manifestations [22]. Defects in any of the numerous mitochondrial pathways can cause mitochondrial diseases and further induce pathological consequences. Accumulating evidence suggests that the variation in the mtDNA of KSS mainly disrupts the respiratory chain function in many tissues, especially in those with a high-energy demand. For example, RPE is a metabolically active cellular monolayer, and a decreased supply of energy can compromise the transportation systems and phagocytosis [10]. As has been previously proven, these novel large-scale deletions often lead to the loss of protein-coding genes or major arc genes of the mtDNA [23], which then affects the polypeptide synthesis of mitochondria and results in an impairment of the oxidative phosphorylation in the patient, which ultimately leads to clinical manifestations of multiple systems. In addition, damage of the mtDNA leads to the ageing process of cells through induction and further effect [24], which can be demonstrated through animal studies. With defective mtDNA polymerase, the mouse showed premature aging as evident in progenitor cells and a malfunction of the proliferative ability of embryonic fibroblasts [25]. In general, spontaneous mtDNA rearrangement has been identified to occur at the germ cell level or in early embryonic development [4]. Moreover, maternal transmission has been reported, and it is thus necessary to examine the mother’s gene of the affected individual for clinical or genetic manifestations of disease [26]. However, in our case, nothing unusual was found in gene detection studies of the patient’s mother, which suggests that our patient was most likely sporadic.

After we confirmed the diagnosis of KSS, we completed the optometry evaluation and provided a pair of applicable glasses, with a necessary follow-up of every 3 months. In addition, further treatment in the Department of Cardiology and Neurology was proposed. To the best of our knowledge, the patient underwent pacemaker implantation. If an ophthalmologist suspects such a disease, early diagnosis can facilitate the optimal management of systemic disease manifestations and may reduce patient morbidity and mortality.

This report has several limitations. The highest heterogeneity of the deletion of mtDNA is often shown in muscle biopsies and with significantly less heterogeneity in blood than in muscle. However, because of the invasiveness of biopsy, the patient’s mother strongly refused the examination. On one hand, ocular electrophysiological abnormalities such as decreased amplitude and prolonged latency may occur in the early stage of RP. On the other hand, diffuse hyperfluorescence in the fundus of the eye can be observed by FFA to determine retinal vascular perfusion and the severity of the disease. However, both of the examinations mentioned above were not performed in this case because of the child’s noncooperation, and neither procedure was performed during the examination.

In summary, we found and reported for the first time a novel large-scale deletion of Chrm.6341–13,993 in a patient with KSS. The newly discovered mtDNA deletion not only makes diagnosis a challenge but also provides stronger evidence for understanding the relationship between mtDNA rearrangement and clinical symptoms. The unique metabolic nature of ocular tissues ensures the leading role of ophthalmologists in the diagnosis and management of patients with mitochondrial disorders. With the development of detection and treatment technologies, growing knowledge is essential, but ophthalmologists are playing an increasingly indispensable role.

## Data Availability

All data generated or analyzed during this study are included in this published article.

## Abbreviations

mtDNA: Mitochondrial DNA
KSS: Kearns–Sayre syndrome
RP: Retinitis pigmentosa
OCTA: Optical coherence tomography angiography
RE: Right eye
LE: Left eye
Cht: Choroidal thickness
MRI: Magnetic resonance imaging
PCR: Polymerase chain reaction
RPE: Retinal pigment epithelium
FFA: Fluorescein fundus angiography
